# Bifunctional Aptamer–Doxorubicin Conjugate Crosses the Blood–Brain Barrier and Selectively Delivers Its Payload to EpCAM-Positive Tumor Cells

**DOI:** 10.1089/nat.2019.0807

**Published:** 2020-03-26

**Authors:** Joanna Macdonald, Delphine Denoyer, Justin Henri, Adelaide Jamieson, Ingrid J.G. Burvenich, Normand Pouliot, Sarah Shigdar

**Affiliations:** ^1^ School of Medicine, Deakin University, Geelong, Australia.; ^2^ Centre for Molecular and Medical Research, Deakin University, Geelong, Australia.; ^3^ Matrix Microenvironment and Metastasis Laboratory, Olivia Newton-John Cancer Research Institute, Melbourne, Australia.; ^4^ Tumour Targeting Laboratory, Olivia Newton-John Cancer Research Institute, Melbourne, Australia.; ^5^ School of Cancer Medicine, La Trobe University, Melbourne, Australia.; ^6^ Department of Pathology, The University of Melbourne, Parkville, Australia.

**Keywords:** aptamer, therapeutic, blood brain barrier, cancer

## Abstract

The prognosis for breast cancer patients diagnosed with brain metastases is poor, with survival time measured merely in months. This can largely be attributed to the limited treatment options capable of reaching the tumor as a result of the highly restrictive blood–brain barrier (BBB). While methods of overcoming this barrier have been developed and employed with current treatment options, the majority are highly invasive and nonspecific, leading to severe neurotoxic side effects. A novel approach to address these issues is the development of therapeutics targeting receptor-mediated transport mechanisms on the BBB endothelial cell membranes. Using this approach, we intercalated doxorubicin (DOX) into a bifunctional aptamer targeting the transferrin receptor on the BBB and epithelial cell adhesion molecule (EpCAM) on metastatic cancer cells. The ability of the DOX-loaded aptamer to transcytose the BBB and selectively deliver the payload to EpCAM-positive tumors was evaluated in an *in vitro* model and confirmed for the first time *in vivo* using the MDA-MB-231 breast cancer metastasis model (MDA-MB-231Br). We show that colocalized aptamer and DOX are clearly detectable within the brain lesions 75 min postadministration. Collectively, results from this study demonstrate that through intercalation of a cytotoxic drug into the bifunctional aptamer, a therapeutic delivery vehicle can be developed for specific targeting of EpCAM-positive brain metastases.

## Introduction

Although the development of new targeted therapies along with improved diagnostic applications has significantly improved progression-free survival of primary malignancies, the estimated incidence of brain metastases is still rising [1]. Similar to primary brain tumors, prognosis of patients with secondary brain lesions is extremely poor, with survival time measured merely in months. Current therapy regimens for brain metastases are multimodal, utilizing a combination of treatment options, including surgical resection, whole-brain radiation therapy, stereotactic radiotherapy, and standard chemotherapy. The efficacy of these treatments is influenced by the number of lesions, their location, age, and health status [2]. In addition, these combinations have shown to improve survival length by only a few months and are associated with a number of debilitating side effects, including neurocognitive dysfunction, nausea, serous otitis media, and hair loss [3,4].

While most primary malignancies metastasize to the brain at the advanced stage of disease progression, triple-negative breast cancer (TNBC) metastasizes during the early stages and can metastasize before primary diagnosis [5,6]. Accounting for approximately 15%–20% of breast cancers, TNBC is characterized by a lack of estrogen and progesterone receptor expression and a lack of human epidermal growth factor receptor 2 overexpression. The absence of these receptors leaves chemotherapy as the mainstay treatment for TNBC patients as they do not respond to targeted treatments currently available for breast cancer [7]. Although chemotherapies have shown to increase survival time, their efficacy is complicated by their indiscriminate and nonspecific nature and development of acquired chemoresistance [8]. Furthermore, the vast majority are ineffective in the treatment of brain metastases due to their poor blood–brain barrier (BBB) penetration and the high expression of multidrug resistance proteins on the BBB [9–11]. Hence, there is a clinical need for development of targeted therapeutic agents for the treatment of TNBC and TNBC brain metastases.

The BBB comprises endothelial cells joined by highly polarized tight junctions and surrounded by astrocytes and pericytes that segregate the brain from the peripheral circulation, impeding the influx of blood-borne molecules that may disturb brain homeostasis [12,13]. The highly restrictive nature of the barrier limits the access of 98% of small molecules to the brain microenvironment, including the vast majority of chemotherapeutics [14]. Overcoming this barrier is the first obstacle in the delivery of chemotherapeutic agents to metastatic brain lesions. A novel approach to achieve this is the development of therapeutic modalities that hijack receptor-mediated transport mechanisms present on endothelial cell membranes. This allows drugs to first pass through the BBB without disrupting it and increases drug delivery through allowing them to circumvent the efflux capacity of the BBB [15].

Given its ubiquitous expression on the BBB, the transferrin receptor (TfR) has been widely exploited for this purpose. The development of therapeutics targeting this receptor has been explored previously using antibodies [16,17]. Yu *et al.* reported on development of a low-affinity antibody directed against the TfR and capable of crossing the BBB to achieve a therapeutically relevant concentration of antibody within the brain in an *in vivo* model. However, given the immunological risk that antibodies pose, novel therapeutic strategies are being explored [16,18].

Nucleic acid aptamers are an emerging field of therapeutics that can provide several advantages over standard antibody-based therapies. Often referred to as chemical antibodies, aptamers are single-stranded oligonucleotides, which bind to their targets through shape recognition, and are therefore amenable to therapeutic applications similar to those intended for antibodies [19]. However, aptamers have the added benefit of a significantly smaller size, lack of immunogenicity, and ease of production [20,21]. One of the many attractive features of aptamers is their ability to be developed as drug delivery vehicles by covalently or noncovalently attaching or linking drug molecules to the aptamer structure. Anthracycline chemotherapeutics, such as doxorubicin (DOX), are widely used in the treatment of TNBC, but their efficacy is poor. Because DOX elicits its cytotoxic effect through direct intercalation into cellular DNA, this property can be exploited to intercalate DOX into the double-stranded region of an aptamer's structure, resulting in the formation of a highly specific drug delivery vehicle. Through intercalation into the aptamer's structure, DOX is specifically delivered and internalized into the targeted cell through receptor-mediated endocytosis. As a result, the indiscriminate side effects of DOX are reduced and the intracellular concentration is increased as drug efflux pumps present on the cell membrane are bypassed. The development of aptamer-DOX conjugates has been extensively reported within the literature [22–24].

We have recently described the generation of a bifunctional aptamer that binds both to TfR and a cell surface receptor, the epithelial cell adhesion molecule (EpCAM), on cancer cells. Preliminary studies demonstrated that this aptamer can cross the BBB and target cancer cells using both *in vitro* and *in vivo* models [25]. In this study, we capitalized on these properties to transform this bifunctional aptamer into a targeted drug delivery vehicle. We developed an aptamer-DOX conjugate using the TfR-EpCAM bifunctional aptamer to demonstrate its suitability for the treatment of TNBC brain metastases. Using a brain-metastatic variant of the MDA-MB-231 mouse model (MDA-MB-231Br), we show for the first time the ability of this system to transcytose the BBB and selectively deliver a cytotoxic payload to targeted tumor cells 75 min postadministration. These preliminary results demonstrate the potential this system has to address the clinical need for targeted treatments for TNBC brain metastases.

## Materials and Methods

### Cell lines and cell culture

The cell lines (mouse brain endothelial cells, bEnd.3; mouse mammary carcinoma cells, 4T1; human embryonic kidney cells, HEK293T; human breast adenocarcinoma cells, MDA-MB-231; and human colorectal adenocarcinoma cells, HT-29) used in this study were purchased from the American Type Culture Collection. Cells were grown and maintained in culture with Dulbecco's modified Eagle's medium (DMEM; Life Technologies) supplemented with 10% fetal calf serum (FCS). The brain-metastatic variant of the MDA-MB-231 human breast cancer cell line (MDA-MB-231Br) was a gift from Prof. Joan Massague (Memorial Sloan Kettering Cancer Center) and cultured in DMEM, 10% fetal bovine serum, sodium pyruvate (1 mM), glutamine (2 mM), and 1% penicillin–streptomycin. MDA-MB-231Br cells were genetically engineered to express turbo GFP and luciferase. All cells were maintained at 37°C in a 5% CO_2_ atmosphere.

### Aptamers

The bifunctional aptamers targeting the TfR and EpCAM (TEPP = TfR positive and EpCAM positive) and the nontargeting control (TENN = TfR negative and EpCAM negative) were purchased from Integrated DNA Technologies (IDT, Coralville, IA) [25]. All oligonucleotide sequences were labeled with a TYE665 fluorophore on the 3′ end and high performance liquid chromatography (HPLC) purified. The sequences were as follows: TEPP: 5′-GC GCG GTAC CGC GC TA ACG GA GGTTGCG TCC GT-3′; and TENN: 5′-GC GCG TGCA CGC GC TA ACG GA TTCCTTT TCC GT-3′.

For experimental procedures, aptamers were prepared at the desired concentration in 5 mM magnesium chloride (MgCl_2_) phosphate-buffered saline (PBS) and folded into their three-dimensional structure using a thermocycler (85°C for 5 min, slow cooling to 22°C over 10 min, and 37°C for 15 min; PerkinElmer).

### Generation of an aptamer-DOX conjugate

To develop the aptamer-DOX conjugates, DOX (44583; Sigma) was conjugated with the folded aptamers in conjugation buffer [0.1 M sodium acetate (CH_3_COONa), 0.05 M sodium chloride (NaCl), and 5 mM MgCl_2_] for 60 min at 37°C under agitation at 75 rpm [26]. The natural fluorescent property of DOX and its subsequent quenching following intercalation allowed efficient measuring of the conjugate molar ratio and loading efficiency. The conjugation molar ratio was determined through incubation of varying aptamer concentrations with a fixed concentration of DOX. The fluorescent signal of varying ratios of the aptamer to DOX (0, 0.01, 0.04, 0.08, 0.1, 0.2, 0.3, 0.4, 0.5, 0.6, 0.7, 0.8, 0.9, and 1) was measured in parallel with a standard curve of DOX using the VICTOR^™^ X5 Plate Reader (PerkinElmer Life).

### Determination of aptamer-DOX binding affinity

The dissociation constant (K_d_) of the aptamer-DOX conjugate was determined by measuring binding to native protein targets using flow cytometric analysis. The cells of interest (5 × 10^5^) were first incubated with binding buffer for 30 min [PBS containing 10% FCS, 0.1 mg/mL transfer ribonucleic acid (tRNA), and 0.1 mg/mL bovine serum albumin], followed by two washes with binding buffer, before incubation with serial concentrations of TYE665-labeled aptamer-DOX conjugates prepared as described in the previous section (0–400 nM) in a 100-μL volume of binding buffer for 60 min at 37°C. The cells were washed three times with PBS, resuspended in 100 μL of PBS, and subjected to flow cytometric analyses (FACS Canto II flow cytometer; Becton Dickinson).

### Cellular uptake and retention of aptamer-DOX conjugates

Twenty-four hours before labeling, MDA-MB-231 and HEK293T cells were seeded at a density of 7.5 × 10^4^ cells/cm^2^ in an eight-chamber slide (LabTekII; Nunc). Following removal of media, cells were incubated in binding buffer at 37°C for 30 min. Cells were then incubated with 1 μM DOX or aptamer-DOX conjugates at an equivalent DOX concentration of 1 μM for 60 and 120 min at 37°C. Bisbenzimide Hoechst 33342 (3 μg/mL; Sigma) was added to the cells during the final 10 min of incubation. Following each time point, the aptamer-DOX and DOX solutions were removed and cells were washed three times for 5 min each in binding buffer before visualization using a FluoView FV10i laser scanning confocal microscope (Olympus). To establish aptamer-DOX retention, following 120 min of incubation, cells were incubated for a further 24 h in DMEM before reimaging. The captured images were then analyzed using ImageJ to quantify DOX fluorescence. Area, integrated density, and mean gray value were measured for cells of interest (15 cells) and background. Corrected total cell fluorescence (CTCF) was calculated using the following formula: CTCF = integrated density − (area of selected cell × mean fluorescence of background readings).

### Cell viability assay

The cytotoxicity of DOX and TEPP-DOX was assessed in MDA-MB-231 cells by determining their half-maximal inhibitory concentrations (IC_50_s). Cells (2 × 10^3^) were plated in a 96-well plate and incubated for 24 h of culture. Subsequently, DOX or TEPP-DOX was added at varying concentrations (0–40 μg/mL DOX diluted in cell culture medium). After 45 h of treatment, thiazolyl blue tetrazolium bromide (MTT; M5655; Sigma) was added to the cells and incubated for 3 h. At the end of the 48-h incubation period, the culture medium was removed and replaced with 150 μL of dimethyl sulfoxide (DMSO). Absorbance (570 nm) was measured using a plate reader (PerkinElmer). The IC_50_ was calculated by nonlinear regression analysis using GraphPad Prism 7.0.

### *In vitro* model of BBB transcytosis of aptamer-DOX conjugates

The insides of Transwell inserts [polyethylene terephthalate (PET) with 0.4 μm diameter pores] within a 24-well plate (COR3379; Corning) were coated with 100 μL of 50% collagen IV (in PBS) for 240 min at 37°C. Wells were filled with 500 μL of DMEM, supplemented with 10% FCS, and Transwell inserts were placed on top. bEnd.3 cells were then seeded onto the luminal side of the filter at a density of 1.34 × 10^5^ cells per filter and were allowed to grow for 6 days. Media in the luminal and abluminal compartments were replaced on days 2 and 4 to supplement the growth of the monolayer. On day 5, media in both compartments were replaced with enhanced media [DMEM:Ham's F12 (1:1), 550 nM hydrocortisone, 32 μM cAMP, 17.5 μM aminophylline, 1 μM retinoic acid, 5 μg/mL insulin, 2.75 μg/mL transferrin, and 2.5 ng/mL sodium selenite]. On day 6, MDA-MB-231 and HEK293T cells were seeded into the lower compartment at a density of 1 × 10^5^ cells per well and allowed to settle for 180 min. Media were then removed from the upper compartments, and 100 μL of aptamer-DOX conjugates at an equivalent DOX concentration of 1 μM was pipetted on top of the Transwell membrane. To differentiate the cell lines, 10 μg/mL of an anti-EpCAM antibody (347197; BD Biosciences) was added to the lower compartment in the last hour of aptamer incubation. Following a 180-min incubation at 37°C, media in the abluminal compartment were removed. Cells (MDA-MB-231 and HEK293T) were then incubated with Bisbenzimide Hoechst (3 μg/mL; Sigma) for 10 min. Cells were then trypsinized and washed three times in PBS before viewing under a FluoView FV10i laser scanning confocal microscope (Olympus).

### Determination of *in vitro* BBB integrity

Barrier integrity was assessed daily through measurement of transendothelial electrical resistance (TEER) using an EVOM 2 (Epithelial Volt/Ohm Meter) resistance meter (World Precision Instruments). The TEER of each Transwell was calculated after subtracting the TEER of a blank Transwell filter and then multiplied by the area of the Transwell to obtain the TEER in Ω cm^2^.

### Brain metastasis assays

All procedures involving mice were performed in accordance with the National Health and Medical Research Council animal ethics guidelines and were approved by the Austin Animal Ethics committee (approval number 17/05429).

MDA-MB-231Br cells (10^5^ cells/100 μL 0.9% saline solution) were inoculated directly into the left cardiac ventricle of 6- to 8-week-old NOD SCID gamma (NSG) female mice, as described previously [27,28]. Mice were monitored daily after cell injection for signs of ill health due to metastasis. From day 7 post-cell injection, the metastatic burden (including in the brain) was assessed weekly by bioluminescence imaging. Briefly, mice were injected intraperitoneally with 200 μL of 15 mg/mL luciferin (Promega) and imaged 10 min after injection using the IVIS *in vivo* imaging system, Lumina II (Xenogen). The tumor burden in organs was assessed using the Living Image software by drawing a region of interest around each organ to allocate animals into treatment groups. Two weeks following cell inoculation, mice with established brain metastases, as determined by bioluminescence imaging, were randomized into four groups (TEPP, TENN, TEPP-DOX, and TENN-DOX, *n* = 3/group) and received a single tail vein injection of treatment (27.5 nmol aptamer; 2 mg/kg DOX). Sixty minutes (TEPP or TENN) or 75 min (TEPP-DOX and TENN-DOX) postinjection, brains were excised and postfixed in 4% PFA, cryoprotected in 20% (wt/vol) sucrose at 4°C overnight, and stored at −80°C [29]. Brains were sectioned (40 μm) on a cryostat and stored in cryoprotectant [30% (vol/vol) glycerol, 30% (vol/vol) ethylene glycol, and 40% (vol/vol) phosphate buffer (PB)] at −20°C. Cryoprotectant was washed off through 3 × 5 min washes in PB containing 0.3% Triton X-100 and a further 2 × 5 min in PB [29]. Sections were mounted onto glass microscope slides and imaged using the FluoView FV10i laser scanning confocal microscope (Olympus).

### Statistical analysis

Results are expressed as mean ± standard error of the mean. Significance (*P* < 0.05) was assessed using the nonparametric Mann–Whitney test using GraphPad Prism 7.0 (San Diego, CA). Unless otherwise specified, all results were averaged from biological triplicates.

## Results

### Development of the aptamer-DOX conjugate

**FIG. 1.**
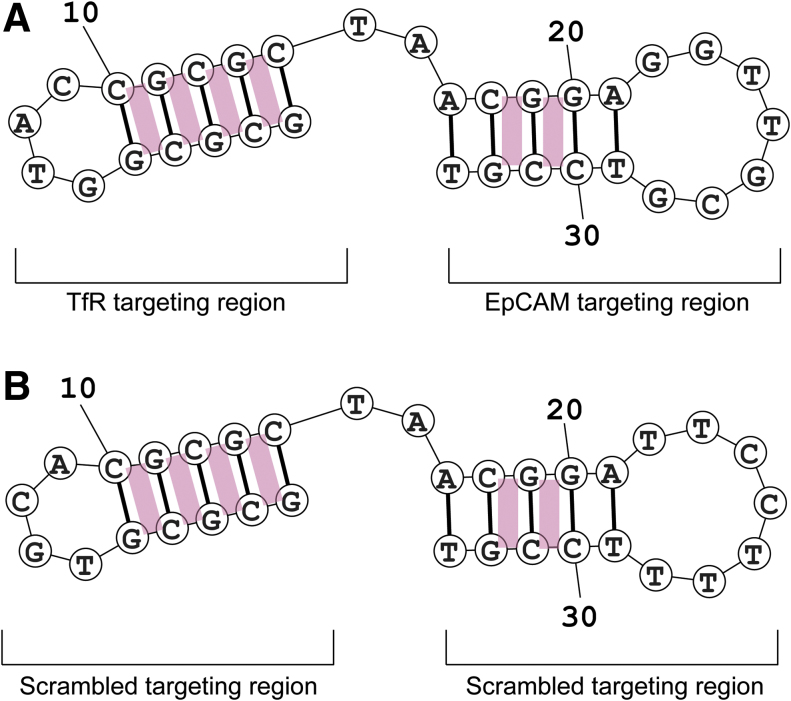
Schematic diagram of the predicted intercalation of DOX in the bifunctional aptamer. **(A)** TEPP aptamer. **(B)** TENN aptamer. Predicted sites for DOX intercalation represented by *pink rectangles*. DOX, doxorubicin. Color images are available online.

To generate a targeted drug carrier capable of transcytosing the BBB and delivering its cytotoxic payload to EpCAM-positive brain metastases, DOX was conjugated with the bifunctional aptamer. The potential sites of DOX intercalation in the bifunctional aptamer were analyzed using the structure prediction software, Mfold [30]. The predicted secondary structure consists of two hairpin structures connected by double-stranded regions. The site of DOX intercalation is between the GC and CG sequences in the double-stranded region of the aptamer, giving the aptamer six potential sites for intercalation (Fig. 1).

**FIG. 2.**
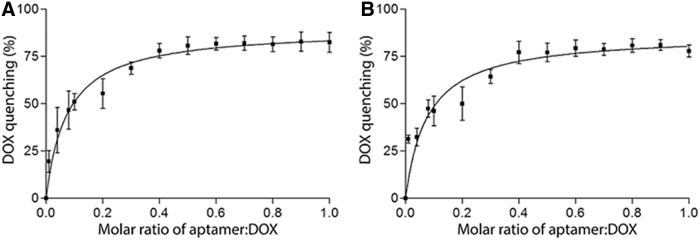
Determination of aptamer-to-DOX molar ratio. The fluorescent quenching of DOX at a fixed concentration (0.6 nmol) with an increasing aptamer-to-DOX molar ratio (0.01, 0.04, 0.08, 0.1, 0.2, 0.3, 0.4, 0.5, 0.6, 0.7, 0.8, 0.9, and 1) was measured after 60 min of incubation at 37°C under agitation at 75 rpm. **(A)** TEPP-DOX and **(B)** TENN-DOX. Data shown are mean ± SEM (*n* = 3). SEM, standard error of the mean.

To determine the optimal molar ratio for loading DOX into the bifunctional aptamer, a conjugation assay was performed with sequential increases of aptamer to DOX. For this, a quantitative assessment was employed, which utilized the fluorescent quenching of DOX subsequent to its intercalation into double-stranded DNA. Shown in Fig. 2, the natural fluorescent signal of DOX was gradually quenched upon intercalation with increasing concentrations of each bifunctional aptamer (TEPP and TENN). The quenching of DOX fluorescence reached a plateau (∼75%) at the molar ratio of 0.4 aptamer to DOX after 60 min of incubation, indicating that approximately two to three molecules of DOX were intercalated per aptamer.

### Preservation of aptamer specificity and selectivity following DOX conjugation

**FIG. 3.**
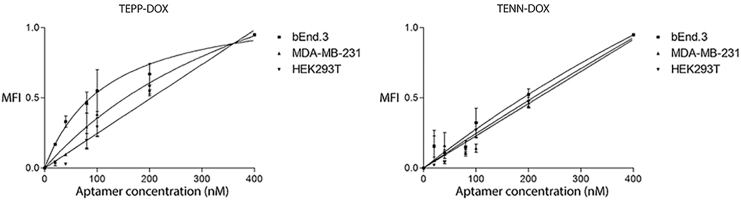
Specificity of bifunctional aptamer-DOX conjugates. TYE665-labeled DOX-conjugated aptamers were incubated with the bEnd.3, MDA-MB-231, or HEK293T cell line and analyzed by flow cytometry. The MFI was plotted against varying concentrations of DOX-loaded bifunctional aptamers (0–400 nM) at a cell density of 5 × 10^5^ cells/mL. Representative binding curves of TEPP-DOX and TENN-DOX with bEnd.3, MDA-MB-231 cells, and HEK293T cells. Data shown are mean ± SEM (*n* = 3). MFI, mean fluorescence intensity.

Introducing modifications or intercalating agents into the stem region of aptamers can lead to a loss of selectivity and/or sensitivity [31]. Therefore, following intercalation, a biological assay utilizing human and mouse cell lines was employed to ensure that the aptamer-DOX conjugates retained specificity and sensitivity to the native conformation of their protein targets. The cell lines used included a cell line expressing the TfR (bEnd.3) and EpCAM (MDA-MB-231) and a cell line expressing neither protein (HEK293T). Following conjugation, TEPP-DOX retained its affinity toward the TfR (K_d_ = 119 ± 30.71 nM vs. K_d_ = 110 ± 22.04 nM for TEPP-DOX and TEPP, respectively), while a decrease in affinity toward EpCAM was observed (K_d_ = 543.5 ± 340.9 nM vs. K_d_ = 85.61 ± 28.49 nM for TEPP-DOX and TEPP, respectively) (Fig. 3 and Table 1). Following DOX intercalation, TENN-DOX showed binding to TfR (K_d_ = 864.5 ± 438.7 nM), but demonstrated no binding toward EpCAM (K_d_ ≥ 10,000 nM). This suggests that DOX intercalation has altered or destabilized the tertiary structure of the aptamer to the point where this aptamer binds weakly to the TfR with low affinity. The intercalation of DOX into these aptamers had no effect on specificity for cells negative for EpCAM and TfR, as determined using the human cell line HEK293T (K_d_ ≥ 10,000 nM) (Table 1).

**Table 1.** Binding Affinity of Bifunctional Aptamer-Doxorubicin Conjugates to the Transferrin Receptor and Epithelial Cell Adhesion Molecule

Aptamer	bEnd.3 (K_d_), nM	MDA-MB-231 (K_d_), nM	HEK293T (K_d_), nM
TEPP	110 ± 22.0	85.6 ± 28.5	>10,000
TEPP-DOX	119 ± 30.7	536 ± 306	>10,000
TENN	>10,000	>10,000	>10,000
TENN-DOX	865 ± 439	>10,000	>10,000

DOX, doxorubicin.

*Source:* Macdonald *et al.* [25].

### Characterization of cellular internalization and retention of aptamer-DOX conjugates

**FIG. 4.**
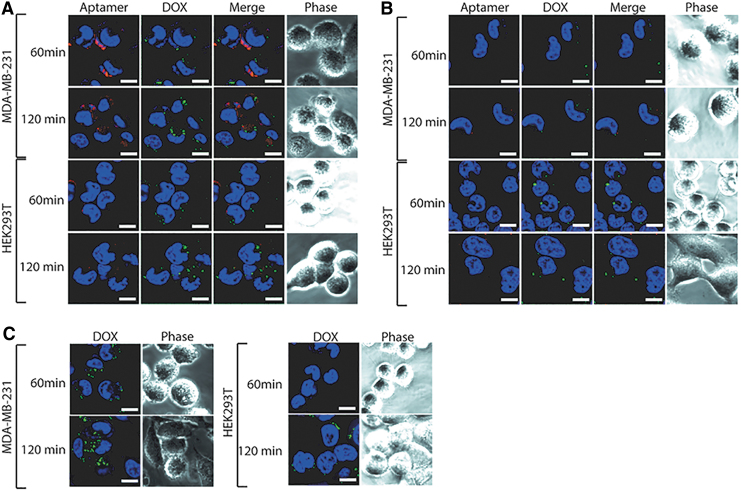
Internalization of bifunctional aptamer-DOX conjugates. Each bifunctional aptamer-DOX conjugate was incubated with EpCAM-positive (MDA-MB-231) or -negative (HEK293T) cell lines at an equivalent DOX concentration of 1 μM for 60 and 120 min at 37°C, followed by visualization using laser scanning confocal microscopy. Representative images of **(A)** TEPP-DOX and **(B)** TENN-DOX. Free DOX **(C)** at a concentration of 1 μM was incubated with EpCAM-positive (MDA-MB-231) and -negative (HEK293T) cell lines for 60 and 120 min at 37°C. *Red*: TYE665-labeled aptamer-DOX conjugates; *green*: DOX; and *blue*: Bisbenzimide Hoechst (3 μg/mL; Sigma). Scale bar: 10 μM (*n* = 3). EpCAM, epithelial cell adhesion molecule. Color images are available online.

With the knowledge that the bifunctional aptamers are internalized intracellularly following 60 min of incubation at a physiologically relevant temperature [25], it was imperative to establish if DOX intercalation affected this ability. To do this, each aptamer-DOX conjugate was prepared at an equivalent DOX concentration of 1 μM and incubated with MDA-MB-231 and HEK293T cells for 60 or 120 min, followed by visualization using laser scanning confocal microscopy. At 60 min of incubation, TEPP-DOX bound to the cell membrane of EpCAM-positive cells (MDA-MB-231) (Fig. 4A) with minimal internalization and DOX release observed, as shown by the fluorescent signal of the aptamer around the perimeter of the cell and lack of DOX fluorescence signal. TENN-DOX showed no binding or internalization (Fig. 4B).

Following an additional 60-min incubation, TEPP-DOX was internalized to varying degrees into the MDA-MB-231 cells, as demonstrated by the red punctate pattern of intracellular distribution indicative of endocytosis (Fig. 4A). The minimal fluorescent signal of DOX detected at this time point indicates that it is yet to be released from the aptamer and that longer than 120 min is required for the drug to be released. Again, TENN-DOX showed no binding or internalization (Fig. 4B). The increase in time for TEPP-DOX to be internalized compared with TEPP indicates that drug intercalation has influenced the internalization rate. After 60 and 120 min of incubation, there was weak fluorescence in the HEK293T cells treated with aptamer-DOX conjugates (Fig. 4A, B) or DOX (Fig. 4C). This suggests low background nonspecific uptake and is consistent with what has been reported for other aptamer-DOX conjugates [32,33].

**FIG. 5.**
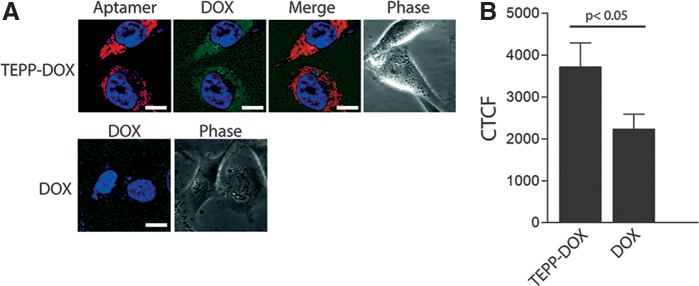
Retention of TEPP-DOX conjugate. To investigate drug retention, following 120 min of incubation with TEPP-DOX or DOX, EpCAM-positive (MDA-MB-231) cells were washed and incubated for a further 24 h in fresh medium. **(A)** Representative images of TEPP-DOX and DOX at 24 h. **(B)** Quantitation of DOX retention at 24 h. Data are representative of three independent experiments. *Red*: TYE665-labeled TEPP-DOX conjugate; *green*: DOX; and *blue*: Bisbenzimide Hoechst (3 μg/mL; Sigma). Scale bar: 10 μM (*n* = 3). CTCF, corrected total cell fluorescence. Color images are available online.

While observing that both aptamer-DOX and DOX were present in the cells following 120 min of incubation, a requirement of drug delivery is that the drug is retained in the cell and is not removed through drug efflux pumps. Therefore, to assess drug retention, MDA-MB-231 cells were incubated with TEPP-DOX or DOX for 120 min and then thoroughly washed and incubated with fresh medium for a further 24 h. Following this period, DOX retained in the cells treated with TEPP-DOX (Fig. 5A) was clearly detectable, whereas only low, residual DOX fluorescence was observed in the cells treated with DOX alone (Fig. 5A). Following quantification using ImageJ, it was established that the DOX fluorescence signal for TEPP-DOX was almost twofold higher than that of DOX (Fig. 5B) at 24 h, indicating superior drug retention. Overall, these data indicate that bifunctional aptamer-DOX resulted in longer retention of DOX in MDA-MB-231 cells compared with DOX alone, similar to our previous study [26].

### Selective cytotoxicity of the TEPP-DOX conjugate to target cells

**FIG. 6.**
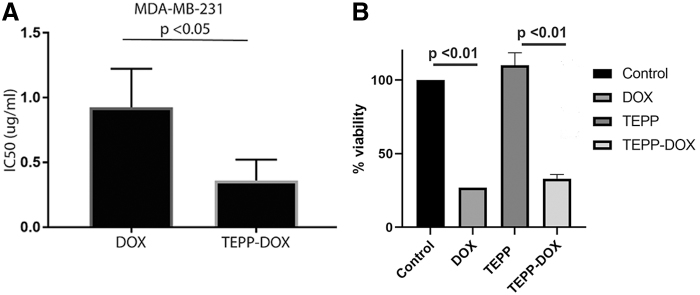
Assessment of DOX, TEPP, and TEPP-DOX cytotoxicity. **(A)** Following 48 h of treatment with DOX and TEPP-DOX (0–40 μg/mL), cell viability was assessed using MTT assay and the IC_50_ value for each treatment was calculated as described in the Materials and Methods section. **(B)** Cell viability for DOX, TEPP, and TEPP-DOX at 10 μg/mL. Data are representative of three independent experiments (*n* = 3). IC_50_, half-maximal inhibitory concentration; MTT, thiazolyl blue tetrazolium bromide.

To examine whether the selective delivery of TEPP-DOX to MDA-MB-231 cells would result in targeted cytotoxicity, the cytotoxic effects of free DOX and TEPP-DOX on MDA-MB-231 *in vitro* were compared. TEPP-DOX was found to be advantageous over DOX alone (Fig. 6). TEPP-DOX demonstrated significantly increased cytotoxicity (2.6-fold) against MDA-MB-231 cells compared with that of DOX alone (IC_50_ TEPP-DOX = 0.362 ± 0.092 μg/mL vs. IC_50_ DOX = 0.927 ± 0.170 μg/mL, *P* < 0.05) (Fig. 6).

### Determination of aptamer-DOX conjugate permeability across an *in vitro* BBB model

**FIG. 7.**
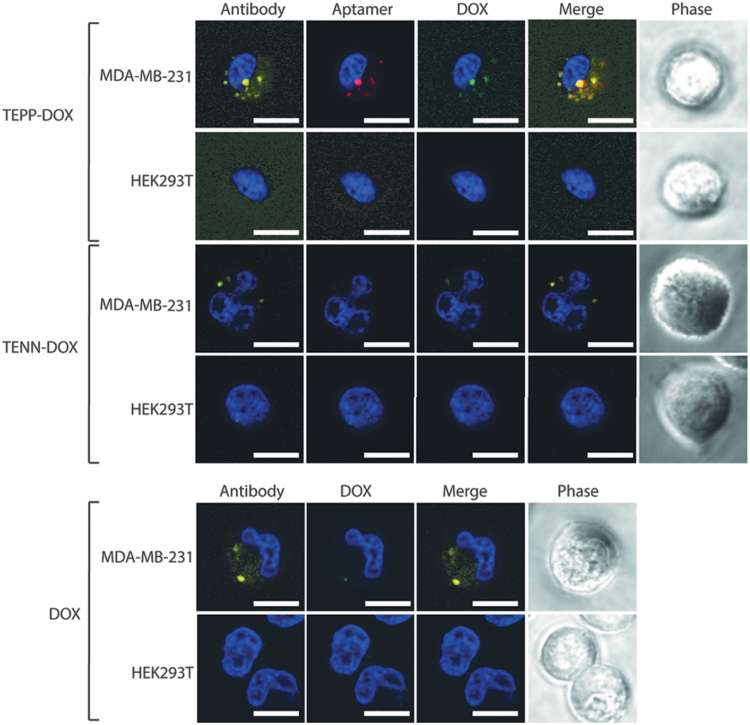
Internalization of bifunctional aptamer-DOX conjugates with an *in vitro* BBB model. Each bifunctional aptamer-DOX conjugate was incubated in the luminal compartment of the *in vitro* BBB for 240 min at 37°C before trypsinization of cells in the abluminal compartment (MDA-MB-231 and HEK293T) and visualization using laser scanning confocal microscopy. An anti-EpCAM antibody was used to differentiate EpCAM-positive MDA-MB-231 cells. Data are representative of three independent experiments. *Yellow*: anti-EpCAM antibody; *red*: TYE665-labeled TEPP-DOX conjugate; *green*: DOX; and *blue*: Bisbenzimide Hoechst (3 μg/mL; Sigma). Scale bar: 10 μM (*n* = 3). BBB, blood–brain barrier. Color images are available online.

To successfully target brain metastases *in vivo*, bifunctional aptamer-DOX conjugates must first be able to cross the BBB. To provide proof of principle that aptamers maintained this ability subsequent to drug intercalation, an *in vitro* BBB model was used. The integrity of the reconstituted barrier was assessed daily, for 6 days, through measurement of TEER. The TEER continued to increase daily, with the highest measurements recorded on day 6, following addition of enhanced medium [DMEM:Ham's F12 (1:1), 550 nM hydrocortisone, 32 μM cAMP, 17.5 μM aminophylline, 1 μM retinoic acid, 5 μg/mL insulin, 2.75 μg/mL transferrin, and 2.5 ng/mL sodium selenite]. The model maintained an average TEER of 35 Ω cm^2^ on day 6, a value consistent with those previously reported for this cell line, indicating sufficient tightness to study permeability [34,35]. To investigate the ability of aptamers to traverse through an endothelial monolayer and to retain functionality and specifically target EpCAM-positive cancer cells, MDA-MB-231 cells were seeded in the abluminal compartment of the *in vitro* BBB model alongside cells devoid of EpCAM expression (HEK293T) at a ratio of 1:1. Each aptamer-DOX conjugate was prepared at an equivalent DOX concentration of 1 μM, added to the luminal compartment, and incubated for 180 min, followed by visualization using laser scanning confocal microscopy. Consistent with the previous findings using the TEPP aptamer [25], TEPP-DOX was able to transcytose through the endothelial monolayer and selectively target MDA-MB-231 cells (Fig. 7). While the TENN-DOX conjugate displayed a low affinity toward the TfR (K_d_ = 864.5 ± 438.7 nM), it failed to internalize into either cell line due to its inability to traverse the endothelial cell layer as previously reported for TENN [25].

### Determination of aptamer-DOX conjugate to transcytose the BBB *in vivo* and specifically target MDA-MB-231Br cells

**FIG. 8.**
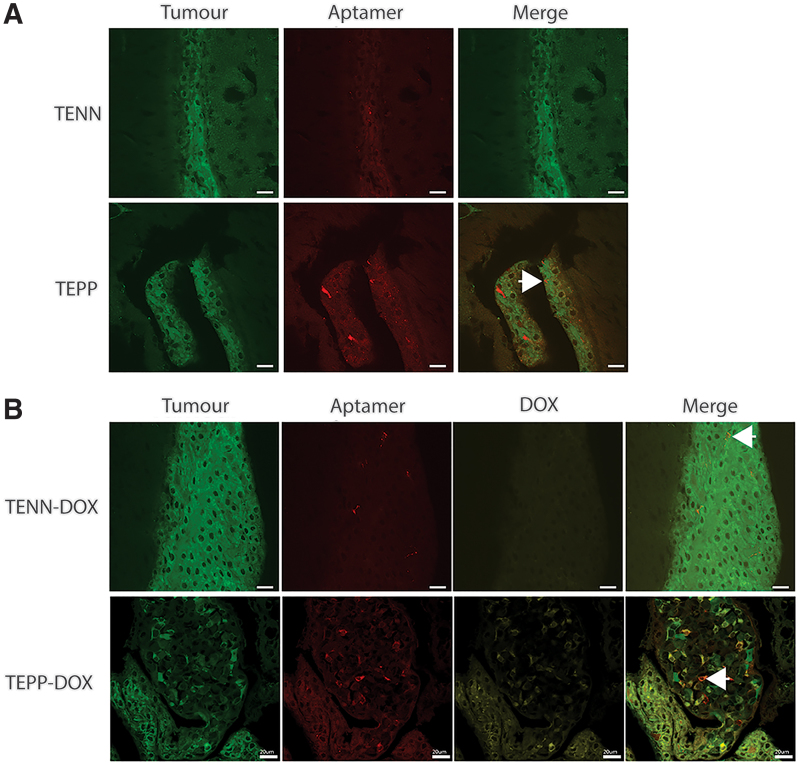
Brain distribution of aptamer or aptamer-DOX conjugates following tail vein injection. Brains were excised 60 and 75 min postintravenous administration, postfixed in 4% PFA, cryoprotected in 20% sucrose at 4°C overnight, sectioned, and imaged using laser scanning confocal microscopy. Representative images of three mice/group. *Green*: GFP tumor; *red*: TYE665-labeled bifunctional aptamer; and *yellow*: DOX. *Arrow* indicates areas of colocalization of aptamer and tumor cell **(A)** or aptamer-DOX and tumor cell **(B)**. Scale bar: 20 μM. Color images are available online.

To demonstrate the ability of TEPP-DOX to target EpCAM-positive, metastatic, breast cancer brain metastases, we made use of the brain-metastatic variant of MDA-MB-231 (MDA-MB-231Br) [36]. The cells were inoculated into the left cardiac ventricle and mice were monitored for development of brain lesions by bioluminescence imaging. Mice with detectable brain metastases were then treated with a single intravenous injection of TENN, TEPP, TENN-DOX, or TEPP-DOX (DOX dose: 2 mg/kg). Sixty minutes (TENN and TEPP) or 75 min postinjection (TENN-DOX and TEPP-DOX), mice were sacrificed and brains harvested, fixed, and imaged. Surprisingly, aptamer was detected in each of the four treatment groups, including TENN and TENN-DOX, most likely entering the brain microenvironment through a nonspecific transport mechanism as reported in a healthy animal model (<0.025% injected dose/g tissue) [25] (Fig. 8), although given that MDA-MB-231Br cells express TfR, some accumulation of TENN-DOX in the tumor might be expected (Supplementary Data and Supplementary Fig. S1). However, there was limited TENN signal colocalizing with the tumor cell population, as indicated by the small amount of colocalization of GFP-positive tumor cells and aptamers (red), further suggesting nonspecific brain uptake. In contrast, the TEPP aptamer clearly colocalized with the GFP-positive tumor cells. This is of significance given that the TEPP aptamer cross-reacts with the mouse receptors, as shown in the EpCAM^-ve^/TfR^+ve^ bEnd.3 endothelial cells (K_d_ 110 nM) (Table 1) or EpCAM^+ve^/TfR^+ve^ murine 4T1 mammary carcinoma cells (K_d_ 27 nM) (Supplementary Fig. S2) *in vitro*, and could compete with binding to human tumor cells in mouse xenograft models. Thus, cross-reactivity of the TEPP aptamer against the mouse receptors provides a more accurate assessment of off-target effects and biodistribution in xenograft models, which will be assessed in future studies. Similar results were observed for the aptamer-DOX conjugates. Interestingly, the uptake of the TEPP-DOX conjugate appeared to be considerably higher than that of unconjugated TEPP, as shown by the high level of intracellular signals in tumor cells.

## Discussion

In this study, we have described the development and characterization of a bifunctional aptamer-DOX conjugate capable of transcytosing the BBB and selectively delivering its cytotoxic payload to EpCAM-positive brain metastases. DOX is one of the most utilized therapeutics in cancer treatment and is commonly employed in the treatment of TNBC [37]. However, due to its cumulative dose-dependent cardiotoxicity and indiscriminate nature, its clinical use is restricted to cumulative doses of 450–550 mg/m^2^ [38]. While in some cases, treatment with DOX can improve the patient survival rate by shrinking tumors and making surgery a viable option, in the case of TNBC brain metastases, it has limited effects due to its inability to cross the BBB [39]. As DOX elicits its cytotoxic effect through preferential intercalation into double-stranded cellular DNA, this property can be exploited to develop highly specific, targeted delivery vehicles using aptamers, which possess a double-stranded DNA stem [26]. We have previously described a bifunctional aptamer that is capable of crossing the BBB and specifically targeting EpCAM-positive cancer cells *in vitro* and that uptake in the brain *in vivo* was superior to control sequences [25]. Thus, we propose that optimal uptake in brain metastases requires cotargeting of both TfR and EpCAM receptors. Given that this small aptamer does not have a cytotoxic effect by itself (Fig. 6B), in this study, we sought to develop this into a targeted therapeutic for the treatment of TNBC brain metastases.

Introduction of DOX into an aptamer's structure has the potential to change the three-dimensional conformation, which in turn can alter the binding interaction between the aptamer and its target [40]. Flow cytometric analysis identified that following intercalation of DOX into the TEPP aptamer, affinity toward the TfR remained largely unchanged, while a large decrease in affinity toward EpCAM was observed (Fig. 3 and Table 1). This observation is consistent with previously reported aptamer-DOX conjugates, whereby DOX intercalation has been shown to both increase and decrease binding affinity [26,41]. As patients with metastatic brain tumors also present with a high extracranial disease burden, the decrease in affinity of the TEPP-DOX-aptamer conjugate toward EpCAM (but not TfR) may be beneficial for the treatment of brain metastases since the aptamer would be expected to preferentially bind to the TfR and increase its bioavailability in the brain tumor. However, this will need to be further investigated in an *in vivo* disease model. If the aptamer binds to systemic metastases preferentially over entering the brain to target brain metastases, a dual injection scheme using the EpCAM aptamer alone to target the extracranial disease burden first could be implemented. This would reduce the available number of EpCAM binding sites systemically, leaving the bifunctional aptamer to target the TfR and transcytose into the brain.

For the cytotoxic payload to be effective, cellular internalization of the aptamer is essential following binding to EpCAM. Fluorescence microscopy (Figs. 4 and 5) revealed that TEPP-DOX aptamer retention was significantly higher than that of DOX alone, although it was not possible to measure the precise concentration of intracellular DOX. The increased DOX signal is likely explained by the differential uptake mechanisms of aptamer-DOX conjugates and DOX alone. Through combining a common chemotherapeutic agent with a target-specific aptamer, drug internalization occurs through receptor-mediated endocytosis rather than free diffusion, turning an indiscriminate drug into a specific one and increasing drug accumulation [42]. Internalization by this method is a crucial factor in the overall design of these drug delivery systems as it plays a major role in drug release. For DOX to be released from the aptamer, protonation of −NH_2_ groups on DOX at a low pH needs to occur [43,44]. This reduces the hydrophobic contact between the aromatic ring of DOX and the DNA bases, leading to drug release [33]. Through being internalized by an endocytic pathway, aptamer-DOX conjugates are exposed to the required pH in the lysosome, resulting in drug release into the cytoplasm, allowing DOX to avoid Pgp pumps present on the cell membrane [45].

Cytotoxic assays highlighted that the cytotoxic effect of the TEPP-DOX conjugate in target cells, MDA-MB-231, was significantly higher than that of DOX alone (Fig. 6). This indicates that once incorporated into the aptamer structure, a targeted delivery system is formed and the concentration of DOX required to elicit a cytotoxic effect is reduced. When compared with a nanoparticle, EpCAM, antibody-based DOX delivery system, the TEPP-DOX cytotoxicity results are significantly superior, with a 300-fold difference in IC_50_s observed [46].

Confirmation of BBB transcytosis following drug intercalation is a pinnacle for the development of this therapeutic modality. To assess if aptamers retained the ability to transcytose the BBB following DOX intercalation and selectively target brain metastases, an *in vitro* model of the BBB was employed [25]. Shown in Fig. 7, drug intercalation did not influence the ability of TEPP to transcytose the BBB and selectively target the EpCAM-positive cell population, consistent with results previously reported for the unconjugated aptamer [25]. While development of bifunctional aptamers has been reported, our study is the first to demonstrate that the DOX-loaded bifunctional aptamer can cross the BBB and deliver a cytotoxic payload. However, while cytotoxicity has been confirmed *in vitro*, it still needs to be evaluated in larger cohorts of mice *in vivo*. The lack of internalization in HEK293T cells further supports the potential ability of the bifunctional aptamer to mitigate the neurotoxic side effects of chemotherapy. Compared with TEPP-DOX, there was no DOX or TENN-DOX detected in either cell population, indicating that they were incapable of transcytosing through the BBB model. While the *in vitro* BBB model is commonly used for assessing BBB permeability, it does not fully replicate the *in vivo* complexity of the BBB, which comprises and is regulated by an intricate balance of a variety of cell types. This complexity is not replicated in an *in vitro* model and is therefore a limitation.

Previous experiments employing healthy *in vivo* models have demonstrated the ability of TEPP to transcytose the BBB, but they have not demonstrated its ability to target a specific cell population [25]. To investigate the ability of TEPP and TEPP-DOX to cross the BBB and selectively target brain metastases, an immunofluorescence assay was conducted whereby mice bearing established brain metastases were treated. Clear distribution of TEPP-DOX was observed in the tumor region, indicating that DOX intercalation had no influence on the aptamer's ability to transcytose the BBB and target EpCAM-positive cancer cells. However, uptake was observed for TENN and TENN-DOX, but showed limited signal in the tumor region. This uptake could be explained by a number of reasons, the first being that a small degree of TENN is taken up nonspecifically in a healthy animal model [25]. Second, during metastatic invasion, the BBB is compromised and thus some aptamers may nonspecifically enter the brain microenvironment at these sites [47]. Additionally, the enhanced permeability and retention effect may be responsible for some accumulation in the tumor [48]. Finally, following DOX intercalation, the TENN aptamer displays an increased affinity to the TfR and theoretically could now traverse the BBB through receptor-mediated transcytosis. As MDA-MB-231Br cells also express TfR (Supplementary Fig. S1), it is possible that TENN-DOX has attached to this receptor in the tumor. However, as there is limited aptamer presence in the tumor, it is likely that due to such a low binding affinity, the aptamer remains close to the BBB or is transcytosed back out of the brain with only a small accumulation in the tumor.

The development of aptamers for treatment of brain cancers and neurological conditions is not a new concept. There have been numerous reports of aptamers for development of treatments for glioblastoma and brain disease [49–51]. However, these aptamers have mainly been suggested for use in diagnostic applications as they lack therapeutic effect. The bifunctional aptamer-DOX conjugate characterized in this study is the first aptamer reported to transcytose the BBB and selectively deliver DOX to tumor cells/brain lesions. Previous methods reported for increasing DOX uptake in the brain have entailed either pre-treatment with agents that interact with multidrug resistance proteins or the use of peptide vectors [52,53]. While these methods resulted in enhanced uptake of DOX, they are limited by the fact that blocking multidrug resistance proteins may lead to uptake of other toxic substances within the bloodstream [51,52]. More importantly, both methods still lack specific delivery upon entering the brain, thus leading to the issue of neurotoxic side effects.

In this study, enhanced uptake into the brain of TEPP-DOX over TEPP may be related to the lower binding affinity of the bifunctional aptamer to EpCAM once DOX has intercalated into the aptamer structure. Conceivably, preferential binding of the TEPP-DOX aptamer through the TfR arm may allow the aptamer to enter the brain preferentially, rather than binding to systemic metastases. This may also affect the biodistribution in other organs. While this study did not investigate biodistribution, our previous study noted accumulation in organs of the reticuloendothelial system [25]. Future studies will investigate if the changed binding affinity has an effect on biodistribution. Additionally, it would be interesting to evaluate if changing the number of DOX molecules intercalated into the aptamer has an effect on binding affinity, biodistribution, and cytotoxicity.

## Conclusions

This study has shown that intercalation of a common chemotherapeutic into a bifunctional aptamer generated a drug delivery vehicle, which is capable of transcytosing the BBB and specifically delivering its payload to EpCAM-positive cancer cells. While previous studies have generated drug delivery vehicles utilizing monofunctional aptamers, this is the first study to explore the use of a bifunctional aptamer as a drug delivery vehicle to target TNBC brain metastases. From previous studies utilizing therapeutic antibodies targeting the TfR, it was discovered that a lower binding affinity was more favorable than a high-affinity antibody for maximal brain uptake, with the highest uptake observed utilizing an antibody with a binding affinity of 111 ± 16 nM [16]. In this study, we have shown that intercalating DOX into the TEPP aptamer generated a drug delivery vehicle with moderate affinity (119 ± 30.7 nM) toward the TfR. Using a physiologically relevant model of disease, we have shown that this delivery vehicle is capable of transcytosing the BBB *in vivo* and specifically delivering its cytotoxic payload to EpCAM-positive brain metastases. Furthermore, the *in vitro* cytotoxicity results in this study indicate that this delivery system could provide better treatment efficacy. Finally, the lack of aptamer-DOX in healthy brain tissue surrounding the tumor suggests increased patient tolerability, resulting in significant improvements in patient quality of life. The ability of this delivery system to mitigate tumor burden and improve overall survival and quality of life is currently being investigated.

## Supplementary Material

Supplemental data
