# Supplementary material for: Bifunctional Aptamer–Doxorubicin Conjugate Crosses the Blood–Brain Barrier and Selectively Delivers Its Payload to EpCAM-Positive Tumor Cells
Source: Nucleic Acid Ther. 2020 Mar 26;30(2):117–28. doi: 10.1089/nat.2019.0807 (PMC7133447; doi:10.1089/nat.2019.0807)
Supplement: Supplemental data [file Supp_Data.pdf]

Supplementary Data

Determination of Transferrin Receptor Expression

Western blotting was performed using a standard methodology, as described previously [S1]. Briefly, 40 µg of proteins was separated on a 4%–12% Bis-Tris gel and transferred to an Immobilon-P PVDF membrane (Merck Millipore). The membrane was blocked with 5% (wt/vol) nonfat dried milk in phosphate-buffered saline (PBS) containing 0.05% Tween-20 for 1 h at room temperature and incubated with an antitransferrin receptor-1 antibody (Cat. No. TFR12-M, 1:1,000 dilution; Alpha Diagnostics) overnight at 4°C. After three washes with the wash buffer (0.05% Tween-20 in PBS) for 15 min, the membrane was incubated for 1 h with an appropriate horseradish peroxidase-conjugated secondary antibody in blocking buffer. Specific protein bands were detected using enhanced ECL reagents and the ChemiDoc™ MP System (Bio-Rad). An anti-GAPDH antibody (Cat. No. Ab8245, 1:10,000 dilution; Abcam) was used as the loading control.

Determination of Aptamer Binding to Mouse Homologs of Transferrin Receptor

The aptamers that form the bifunctional aptamer were generated to target mouse transferrin receptor (TfR) and human epithelial cell adhesion molecule (EpCAM). To assess the translatability of the bifunctional aptamer, we performed a preliminary binding assay to determine if the aptamer would bind to the protein homologs of TfR in an additional mouse cell line. Binding assays were performed, as previously described, using the 4T1 murine mammary carcinoma cell line, an EpCAM/TfR-expressing cell line [S2]. As seen in Supplementary Figure S2, TEPP shows binding to 4T1 cells, while TENN does not. The moderate binding affinity of TEPP to 4T1 cells, of 27.6 ± 20.1 nM, suggests that this aptamer could be used in future preclinical models to determine off-target effects in a clinically relevant immune competent model of breast cancer brain metastases [S3]. TENN binding affinity for 4T1 was very low (>5,000 nM).

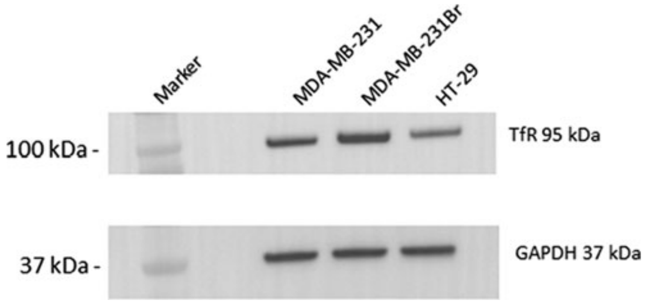

SUPPLEMENTARY FIG. S1. Western blot analysis of transferrin receptor 1 expression in MDA-MB-231, MDA-MB-231Br, and HT-29 cell lines.

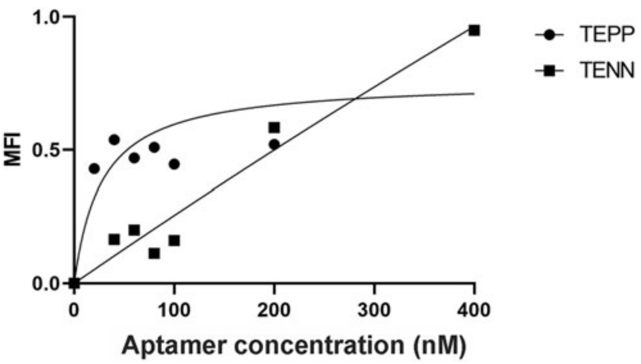

SUPPLEMENTARY FIG. S2. Specificity of the bifunctional aptamers. TYE665-labeled aptamers were incubated with the 4T1 cell line and analyzed by flow cytometry. The MFI was plotted against varying concentrations of bifunctional aptamers (0–400 nM) at a cell density of 5 × 105 cells/mL. Representative binding curves of TEPP and TENN with 4T1 cells. Data shown are mean ± SEM ($n = 1$). MFI, mean fluorescence intensity; SEM, standard error of the mean.

Supplementary References

S1. Denoyer D, N Kusuma, A Burrows, X Ling, L Jupp, RL Anderson and N Pouliot. (2014). Bone-derived soluble factors and laminin-511 cooperate to promote migration, invasion and survival of bone-metastatic breast tumor cells. Growth Factors 32:63–73.

S2. Jenkins SV, ZA Nima, KB Vang, G Kannarpady, DA Nedosekin, VP Zharov, RJ Griffin, AS Biris and RPM Dings. (2017). Triple-negative breast cancer targeting and killing by EpCAM-directed, plasmonically active nanodrug systems. NPJ Precis Oncol 1:27.

S3. Kim S-H, RP Redvers, LH Chi, X Ling, AJ Lucke, RC Reid, DP Fairlie, ACBM Martin, RL Anderson, *et al.* (2018). Identification of brain metastasis genes and therapeutic evaluation of histone deacetylase inhibitors in a clinically relevant model of breast cancer brain metastasis. Dis Model Mech 11:DMM034850.
